# Factors Associated With Successful Parathyroid Adenoma Localisation in Sestamibi Study—Can Change in Serum Calcium Be a Useful Indicator?

**DOI:** 10.1155/ije/3922886

**Published:** 2025-09-12

**Authors:** Peter Jarvis, Jennifer Downs, Tony Skene, Abigail Evans, Tristan Richardson, Amit Parekh

**Affiliations:** ^1^Radiology Department, University Hospitals Dorset NHS Foundation Trust, Poole, UK; ^2^Department of Medical Science & Public Health, Bournemouth University, Poole, UK

**Keywords:** calcium, hyperparathyroidism, localisation, parathyroid, sestamibi

## Abstract

**Objective:** Primary hyperparathyroidism can be cured by the successful removal of the culpable parathyroid adenoma. Successful localisation allows the specialist surgeon to perform safer and more cost-effective focused excisions rather than exploratory surgery. This study aims to identify possible factors that predict successful adenoma localisation using technetium99m–sestamibi.

**Design, Patients and Measurements:** Retrospective analysis of 159 patients undergoing parathyroid localisation with technetium99m–sestamibi SPECT/CT. Patients were classified as successful or unsuccessful localisation when compared to the surgical site of a proven adenoma following successful parathyroidectomy. Preoperative and postoperative serum parathyroid hormone (PTH), calcium and 25-hydroxyvitamin D levels and pathological size of the parathyroid adenoma were recorded.

**Results:** Larger specimen volume, weight and higher preoperative PTHs were strongly associated with successful localisation. The percentage change in serum calcium (calculated as the difference between pre- and post-op calcium) was also strongly associated with successful localisation. Higher preoperative serum calcium (> 2.85 mmol/L) was also associated with successful localisation although with a reduced statistical significance. Seventy percent of patients in our cohort underwent parathyroidectomy with a serum calcium < 2.85 mmol/L, of which 92% had pathologically confirmed adenomas and 67% had successful localisation with sestamibi.

**Conclusion:** The serum PTH and change in serum calcium were most strongly associated with successful localisation. The degree of hypercalcaemia was also associated with successful localisation but without as strong an association when compared to the change in calcium. Several factors influence the degree of hypercalcaemia in patients with primary hyperparathyroidism including parathyroid adenoma size, 25-hydroxyvitamin D status and the individual's baseline calcium set point. Historic information (if available) on the patient's individual baseline set point prior to developing primary hyperparathyroidism, and subsequent elevation when primary hyperparathyroidism has developed, could aid decision-making for clinicians when deciding on parathyroidectomy.

## 1. Introduction

Primary hyperparathyroidism is a common endocrine disorder with a prevalence of 1–4/1000 in the United Kingdom [1]. It is caused by overactivity of one or more parathyroid glands most commonly through the development of a noncancerous adenoma. Patients can be asymptomatic, display symptoms related to high serum calcium (fatigue and bone aches) and/or develop complications such as renal stones or osteoporosis [2].

Primary hyperparathyroidism is cured by the surgical removal of culpable parathyroid adenomas/hyperplastic glands. Detection and accurate localisation of the parathyroid adenoma allows dedicated specialist surgeons to perform a focused excision rather than full four-gland exploratory surgery, which has a longer operative time and recovery and higher risk of postoperative complications [3].

Several investigations can be performed to localise an adenoma with varying sensitivity, specificity, cost and radiation exposure. Most centres use a combination of ultrasound and sestamibi nuclear medicine studies [4]. Newer 4D CT and PET/CT techniques are available as further aids to localisation [5]. In our centre, we primarily use ultrasound and dual time point technetium99m–sestamibi with single photon emission computer tomography with CT (SPECT/CT).

Historically, the degree of hypercalcaemia is often used to help determine surgical intervention (and therefore, which patients will require localisation studies [1]). National and international guidelines have a serum adjusted calcium value greater than 2.85 mmol/l as an isolated biochemical indication for parathyroidectomy in primary hyperparathyroidism. One of the reasons for this recommendation was to recognise that as serum calcium becomes more elevated, there is a greater chance of acute hypercalcaemia-related symptoms and a greater risk of hypercalcaemic crisis, particularly if the patient becomes unwell or dehydrated, rather than a link to symptoms or complications. However, many centres misinterpret this as a cut-off for consideration of surgery in asymptomatic, uncomplicated patients with primary hyperparathyroidism. This may reduce access to curative parathyroidectomy for patients with adjusted calcium concentrations < 2.85 mmol/L (authors' experience), who are deemed to have more mild degrees of hypercalcaemia.

Many historic international guidelines set a degree of hypercalcaemia of > 2.85 mmol/L based on the premise that 2.85 mmol/L was 0.25 mmol/L above the upper limit of normal for most reference ranges for calcium [6]. We hypothesise that patients with a calcium < 2.85 mmol/L may still have had a consequential rise in calcium from their set baseline and still have significant hyperparathyroidism despite lesser degrees of hypercalcaemia overall (and may still have acceptable localisation rates and thus access lower risk parathyroidectomy). Consideration for parathyroidectomy (and therefore localisation studies) should still be entertained in these patients as appropriate—particularly if there is a notable change from the individual's baseline set point calcium, such as an increase of 0.25 mmol/L above individual baseline if known.

There are several prior studies that have demonstrated a direct positive correlation between a high serum PTH [7–9] or a high serum calcium [8, 10] and successful sestamibi localisation in patients with known primary hyperparathyroidism. A few studies have looked at the association of 25-hydroxyvitamin D levels on the success of sestamibi localisation [11, 12], but none to our knowledge have investigated any correlation with the change in calcium in relations to successful localisation. There is a general lack of consensus in the literature about which factors are associated with parathyroid localisation on sestamibi SPECT/CT.

## 2. Methods

Retrospective analysis was performed on 195 patients undergoing parathyroid adenoma localisation with dual time point washout technetium 99m sestamibi SPECT/CT (sestamibi scintigraphy) for primary hyperparathyroidism between January 2018 and April 2021 at a single UK centre. Surgical and pathological data were analysed for adenoma localisation and characteristics. Pathological specimen data were used to confirm successful adenoma identification. Preoperative sestamibi scans were reviewed on Picture Archiving and Communications System (PACS) by a radiologist with 4 years' experience and access to the original consultant report. The radiological site and surgical site of the adenoma were compared to determine if localisation was deemed successful, taking surgical site as the gold standard. For the purposes of this study, successful localisation was determined when the correct side (left or right) on sestamibi scintigraphy matched with the surgical site. If there was an upper or lower pole discrepancy, but the correct side was identified, then it was determined to be a successful localisation. Preoperative serum PTH, calcium and 25-hydroxyvitamin D results were obtained from electronic patient records.

Twenty patients had no surgical data available and were therefore excluded from the study. Twelve patients with no adenoma identified at surgery (1 false positive, 11 true negative) and one patient with papillary carcinoma of the thyroid were excluded from the analysis.

The adenoma was presumed to be ellipsoid in shape with the volume calculated using the following formula:

(1) $$\begin{array}{c} \text{Volume}=\frac{4}{3}\times \pi \times A\times B\times C, \end{array}$$

where *A*, *B* and *C* are the lengths of all 3 semiaxes of the ellipsoid.

Statistical analysis was performed using Mann–Whitney U and linear regression. Chi square and *t*-test were used for demographic data with a *p* value of < 0.05 deemed statistically significant.

## 3. Results

Overall, 159 patients were available for final analysis and divided into those with successful adenoma localisation (*n* = 112) and unsuccessful localisation (*n* = 47). Patient demographics were similar between both groups (Table 1).

Of the 159 patients, all had preoperative PTH and calcium levels available, 134 had weight recorded on pathological data and 128 patients had sufficient data available to calculate an ellipsoid volume of the adenoma. One hundred and thirty-four patients had a 25-hydroxyvitamin D level recorded within 6 months prior to the sestamibi scan. Nine patients did not have postoperative calcium available to be able to calculate a change in serum calcium.

There were 3 cases where 2 adenomas were detected at surgery. In these cases, the volume and weight of both specimens have been included separately and correlated independently to localisation on the sestamibi scan. If the sestamibi scan was unable to correctly identify both adenomas when 2 were found at surgery, then this was determined an unsuccessful localisation with regards to preoperative PTH, calcium and 25-hydroxyvitamin D analysis.

**Figure 1:**
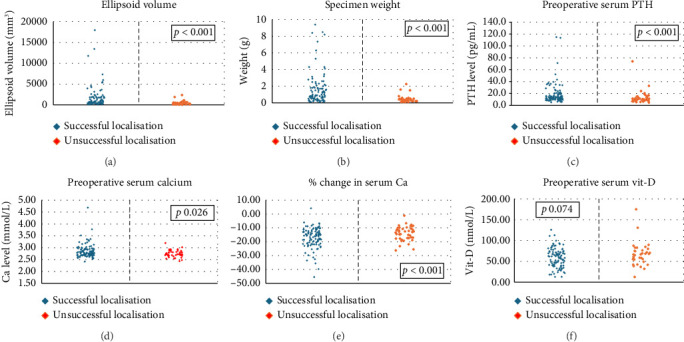
Scatter bar charts comparing successful and unsuccessful sestamibi localisations with (a) pathological specimen volume, (b) pathological specimen weight, (c) preoperative serum PTH, (d) preoperative serum calcium, (e) percentage change in serum calcium postoperatively and (f) preoperative serum vitamin D. Successful sestamibi localisations had greater volume and weight and higher preoperative serum PTH and Ca. There was a greater percentage change in serum Ca with successful localisations. There was no significant difference in preoperative serum Vit-D between successful and unsuccessful localisations. Abbreviations: PTH = parathyroid hormone, Ca = calcium and Vit-D = 25-hydroxyvitamin D.

**Figure 2:**
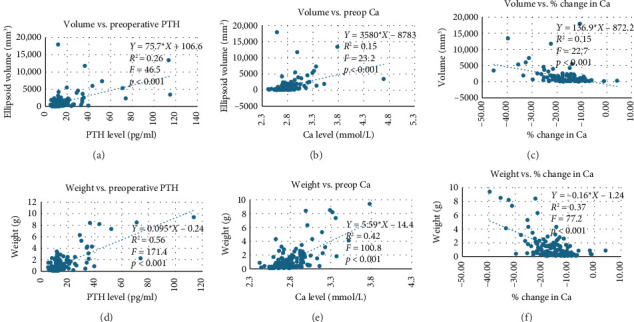
Scatter plots comparing ellipsoid volume with (a) preoperative serum PTH, (b) preoperative serum Ca and (c) % change in serum Ca and specimen weight with (d) preoperative serum PTH, (e) preoperative serum Ca and (f) % change in serum Ca. There are positive correlations with serum PTH and Ca with specimen volume and weight. Similarly, the larger the % change in serum Ca, the greater the specimen volume and weight. Abbreviations: PTH = serum parathyroid hormone and Ca = serum calcium.

Results are shown in Tables 2 and 3 and Figures 1 and 2.

Larger mean specimen volume (1177 mm^3^ vs. 451 mm^3^, *p* < 0.001) and weight (1.69 g vs. 0.43 g, *p* < 0.001) were associated with successful sestamibi localisation (Figures 1(a) and 1(b)). Similarly, a higher mean preoperative PTH level (18.0 pg/mL vs. 12.8 pg/mL, *p* < 0.001) was associated with successful localisation (Figure 1(c)). Higher mean preoperative calcium levels (2.85 mmol/L vs. 2.75 mmol/L, *p* = 0.026) were also associated with successful localisation (Figure 1(d)) but with a lower level of statistical significance. We found that the mean percentage change in serum calcium pre- and postoperatively had a greater statistically significant association with successful localisation (Figure 1(e): −17.5% vs. −14.1%, *p* < 0.001) than preoperative calcium alone. Mean serum 25-hydroxyvitamin D levels measured within 6 months prior to the sestamibi study were lower in those with successful localisations but did not reach the threshold for statistical significance (Figure 1(f): 58.5 nmol/L vs. 67.0 nmol/L, *p* = 0.074). Sixty-six percent (88/134) of patients in our cohort had an initial 25-hydroxyvitamin D level > 50 nmol/L. Subsequent 25-hydroxyvitamin D replacement for all patients with primary hyperparathyroidism preoperatively has been part of routine practice since 2005.

We found positive correlations with both ellipsoid volume (*p* < 0.001) and weight (*p* < 0.001) with preoperative PTH and preoperative calcium. Similarly, an inverse correlation was seen with ellipsoid volume (*p* < 0.001) and weight (*p* < 0.001) with a change in calcium (Figure 2).

One of the parameters included in the NICE recommendations for consideration of parathyroidectomy in asymptomatic primary hyperparathyroidism includes preoperative calcium 2.85 mmol/L or above [1]. Of all the 171 patients that underwent surgery, 70% (119/171) had preoperative calcium < 2.85 mmol/L, of which 92% (109/119) had a pathologically confirmed adenoma removed. Of the patients with adenomas present, 67% (73/109) had successful sestamibi localisation. Similarly, in the 30% (52/171) of patients with a preoperative calcium ≥ 2.85 mmol/L, 96% (50/52) had a pathologically confirmed adenoma removed and 82% (41/50) were successfully localised (Table 3). The difference between successful localisations between these 2 groups did not quite reach statistical significance (*X*^2^ = 3.8, *p* = 0.051).

## 4. Discussion

Parathyroid adenoma size, serum PTH and change in calcium levels had a clear association with successful parathyroid adenoma localisation (Figures 1(a), 1(b), 1(c), 1(e)). Higher preoperative calcium was also associated with successful localisation (Figure 1(d)); however, this was a less clear association than the change in calcium.

This study corroborates the findings of previous studies that larger adenomas are more likely to be correctly identified on sestamibi SPECT/CT [13]. This is due to the predilection of sestamibi to concentrate within cells containing high mitochondrial content, with larger adenomas containing more cells concentrate a larger amount of sestamibi [14, 15]. Our study also highlights the relationship between larger adenomas and higher levels of PTH (Figure 2(a)), supporting the principle that adenomas accompanied by higher PTH levels were more likely to be localised due to parathyroid adenoma size.

In patients with primary hyperparathyroidism, calcium and PTH can be influenced by several factors including parathyroid adenoma size, 25-hydroxyvitamin D status, but also the calcium ‘set-point' for that individual within the reference range of 2.2 mmol/L–2.6 mmol/L. This reference range is reflected by a Gaussian curve with near normal distribution [16] with the expectation that 50% of patients would have a baseline calcium set-point under 2.4 mmol/L. We hypothesise that some parathyroid adenomas will develop in patients with lower baseline calcium set-points but still be associated with a significant calcium rise (with or without associated symptoms and end organ disease), however without necessarily developing frank hypercalcaemia. This could be seen in normocalcaemic hyperparathyroidism, where a patient with low-normal calcium can continue to have a calcium level within the normal assay range when primary hyperparathyroidism manifests [17]. As an example, a patient with known starting baseline calcium of 2.2 mmol/L developing an adenoma with calcium rising to 2.65 mmol/L has a change in calcium of 0.45 mmol/L (but serum calcium level is only 0.05 mmol/L above the normal reference range). This would be equivalent to a patient with baseline calcium of 2.5 mmol/L, developing primary hyperparathyroidism with a similar sized adenoma and rise in calcium of 0.45 mmol/L to 2.95 mmol/L (however, now serum calcium is 0.35 mmol/L above the normal reference range). Our study shows that the change in calcium pre- and postoperatively had a stronger association than preoperative calcium alone for successful adenoma localisation, thus providing evidence for this hypothesis. As far as we are aware, this has not previously been published in the literature.

With the general increased frequency of blood testing in primary care [18], there may be more availability of historic calcium levels, which could be a very useful baseline or set-point calcium for that individual. This can then be used to calculate a “surrogate change in calcium” when primary hyperparathyroidism has developed.

One of the NICE recommendations is that asymptomatic patients with preoperative corrected calcium of 2.85 mmol/L or greater should be considered for surgery [1]. We suggest, in addition to this criterion, the change in the calcium level may add useful information in guiding treatment. In our study, 70% of patients referred for surgery had preoperative calcium less than 2.85 mmol/L, of which 67% had successful sestamibi localisation and 92% had successful surgery (Table 3). Our study advocates that in addition to using preoperative calcium to determine intervention, consideration of patients who have had a significant rise in calcium from their baseline set-point (e.g. > 0.25 mmol/L) within the reference range, would also be good candidates for successful localisation, and therefore successful minimally invasive parathyroidectomy.

This was a retrospective analysis and is therefore subject to gaps in the data, such as incomplete pathological size measurements. Whilst all studies were second read, correlated with the original consultant report and initially blinded to pathological results, there remains the possibility of bias from the retrospective nature of this study.

A further limitation comes from the inherent uncertainty when reporting sestamibi studies. In several instances, the report did not mention a definite adenoma but did raise the possibility of an adenoma based on some mild nonspecific tracer retention. When such uncertainty arose, we chose to record the study as positive adenoma identification. This may lead to over-representation of true positives in this study. This population group also demonstrated an unusual proportion of single adenomatous disease.

Localisation of disease as ‘superior gland' and ‘inferior gland' is notoriously difficult due to the propensity for enlarged superior glands to move inferiorly in the neck [19]. Parathyroid location also remains variable and therefore superior versus inferior localisation has been excluded from this study.

Lastly, this is a very heterogeneous cohort of patients with varying severity of disease that could influence interpretation of blood results. There were varying degrees of 25-hydroxyvitamin D deficiency in the cohort as expected from any retrospective study of a clinical service. The potential confounding effect of varying 25-hydroxyvitamin D levels may be ameliorated by our standard of routine 25-hydroxyvitamin D replacement in this population.

## 5. Conclusion

This study identified that PTH and the change in calcium had clear associations with successful parathyroid adenoma localisation, likely due to their significant correlation with adenoma size. The change in calcium showed a greater degree of statistical significance for successful localisation than preoperative degree of hypercalcaemia alone. Absolute degree of hypercalcaemia was also correlated with successful localisation.

When selecting patients for parathyroid localisation and surgery, we propose that, where available, a calculated significant change in calcium (for example of > 0.25 mmol/L from baseline) could be a useful additional indicator. This could have a role alongside the multiple factors already taken into consideration, such as the cut-off preoperative calcium of 2.85 mmol/L already used.

## Figures and Tables

**Table 1:** Patient demographics.

Variable	Successful localisation	Unsuccessful localisation	*p* value
Median age (years)	60 (22–85)	58 (33–79)	0.37
Gender (% female)	70 (78/112)	77 (36/47)	0.37

**Table 2:** Summary of factors associated with successful adenoma identification on washout sestamibi scan.

	Adenoma found	Failed to identify adenoma	*p* value (Mann–Whitney *U*)
Mean specimen volume (mm^3^)	1767	451	< 0.001
Mean specimen weight (g)	1.69	0.43	< 0.001
Mean preoperative PTH level (ng/mL)	18.0	12.8	< 0.001
Mean preoperative calcium level (mmol/L)	2.85	2.75	0.026
% change in serum calcium	−17.5	−14.1	< 0.001
Preoperative 25-hydroxyvitamin D level (pmol/L)	58.5	67.0	0.074

Abbreviation: PTH = parathyroid hormone.

**Table 3:** Number of patients with successful localisation and surgery with serum Ca below and above 2.85 mmol/L.

	Number in cohort	Successful localisation	Successful surgery
Ca < 2.85 mmol/L	119	73 (67%)	109 (92%)
Ca ≥ 2.85 mmol/L	52	41 (82%)	50 (96%)

*Note:* Ca = corrected serum calcium.

## Data Availability

Data can be obtained from the corresponding author on request.
